# Case of Hysterical Catalepsy; with Remarks

**Published:** 1827-11

**Authors:** John North

**Affiliations:** Surgeon Accoucheur.


### HYSTERICAL CATALEPSY.

*Case of Hysterical Catalepsy; with Remarks.* By JOHN NORTH, Surgeon Accoucheur.

CATALEPSY is a disease of such unfrequent occurrence that many highly respectable authorities, and CULLEN amongst the number, have altogether denied its existence. The few instances upon record were regarded as examples of credulity on the part of the practitioner, and imposition on that of the patient. It must be confessed, indeed, that many of the phenomena which have been said to accompany this species of nervous derangement are so very extraordinary, that the total scepticism of those who have not themselves seen an instance of the disease may easily be pardoned. The case, of which I am about to give a brief description, was not attended by any of those marvellous symptoms which have been recorded by some writers.* It exhibited, however, many curious changes, and may not be altogether uninteresting as an additional illustration of the variable character of diseases which principally affect the nervous system, particularly when they occur in highly susceptible females.

The impossibility of assigning either a fixed place or title to such diseases in nosological arrangements, must also be sufficiently evident. Hence the various terms which have been applied to this form of complaint by different nosologists. The tendency which all nervous diseases have to pass into each other is well known; and in many other instances, besides that I am about to relate, I have seen such a variation

* Dict. des Sciences Med. p. 282, tome iv.

of symptoms in hysterical women in the course of a single day, as would have induced different practitioners to have considered the disease to be either chorea, hysteria, epilepsy, apoplexy, lethargy, or tetanus, according to the particular train of symptoms that happened to exist at the moment of their watching the patient.

Some cases of catalepsy have been related which occurred in men of a highly irritable temperament, but the disorder is certainly much more frequent in females. The same observation will perhaps apply with equal propriety to all convulsive diseases. The different symptoms which are produced in women of a very nervous and susceptible constitution, by either mental or corporeal excitement, must be referred rather to individual peculiarity, than to a corresponding difference in either the predisposing or exciting causes.

Case.—Eliza T., aged twenty-one, had enjoyed a tolerably good state of health up to the period of her present attack. She had always been of a very irritable temper, and occasionally had suffered much inconvenience from severe paroxysms of hiccup, which were presumed to be dependent upon an habitual constipation of the bowels. It was not uncommon for her to pass a week without any evacuation, while in other respects she appeared to be in perfect health. She menstruated regularly, and, from the information I received from her friends, the discharge was of the usual quantity. In the beginning of last March she arrived in London, much fatigued by a long journey, and I have reason to believe in a state of great mental anxiety, on account of some attachment she had formed in the country. A short time after her arrival, she was attacked with pain and swelling of her foot. This was considered to be a rheumatic affection, and was treated accordingly. She then complained of intense pain in the head, and had for the first time slight hysterical paroxysms. She was bled and blistered, and purgatives were freely administered. Her head was also shaved, and cold lotions constantly applied. Each day presented such a transition of her symptoms as to excite some doubt as to the real nature of her complaint, and the opinion of an eminent physician was taken, who regarded the case as altogether hysterical.

In consequence of the removal of the patient to the house of a friend who resided in my neighbourhood, I was sent for to see her on the 30th of March. My attendance was requested as speedily as possible, as she was supposed to be in a dying state. I found her in bed, and apparently in a profound sleep. She had just fallen into this state after a violent fit of hysterics. Not the slightest motion could be observed in any part of her body. No pulsation at the wrist could be felt. The action of the heart was so extremely feeble, that it was distinguished with difficulty; and no motion whatever could either be seen or felt in the carotids. The pupil of each eye was contracted to a very minute point. The temperature of the body was below its natural degree. A glass was held before her mouth, but no vapour could be detected on its surface. Her respiration was not perceptible, for the chest appeared as perfectly free from motion as the other parts of the body. Various stimuli had been applied without effect. I remained with her about an hour, and perceived that she drew a deep but gentle inspiration about every ten minutes, and that there was an occasional slight trembling motion of the eyelids. I relieved the anxiety of the friends by assuring them that the patient would rally from this state, and directed the application of mustard sinapisms to the feet. A stimulating enema was also administered, and I placed four drops of croton oil upon the tongue.

In this state she continued for about twelve hours, when a slight hysterical paroxysm came on; after which she rose languidly from her bed, and complained only of great weakness and pains in different parts of her body. She was perfectly unconscious of every thing that had occurred.

For two days she appeared rather better. Her strength was increasing, and the hysterical attacks were neither so frequent nor so severe. The croton oil acted powerfully upon her bowels. Light tonics were now prescribed, and she was directed to be kept as quiet as possible, both in mind and body.

On the third day, she was suddenly attacked with so violent a trembling of her whole frame as to shake the bed on which she lay. In about half an hour she became perfectly tranquil, and appeared as if she were sleeping. A very violent attack of hysteria, attended with general convulsions, now came on, and it required the exertion of several persons to prevent her from injuring herself. The enema and sinapisms were repeated as before, and purgatives were given by the mouth as soon as possible, as the bowels had again become torpid. As she had not passed any water for nearly twenty-four hours, the catheter was introduced, but only about three or four ounces of very high-coloured urine were drawn off. A copious evacuation of dark-coloured fæces was discharged from the bowels; and on the following day, when I saw her, she complained of nothing more than great languor, and an indisposition to any exertion.

In a few hours after I had left her, she fell into a state of complete stupor, during which the limbs and body appeared alternately under the influence of the nervous power. First the arms were completely extended, and thrown into various positions. The legs were then gradually raised upwards, and remained at their full stretch without support. When these parts became relaxed, the body and head were affected in a similar manner. The attack now assumed all the characters of catalepsy. She resembled a figure of wax, which might be moulded to any form. In whatever position she was put, she remained immoveable as a statue, however awkward and fatiguing it might be. She was, for instance, placed in a sitting position, with her arms in a boxing attitude, and thus she remained until the caprice of the bystanders put her into some other form. One eye was opened to its full extent, the other being at the same moment closed. It remained fixed, and the pupil as perfectly contracted and immoveable as before. The globe of the eye appeared quite insensible to the touch, as did the other parts of her body. On two or three occasions, when the whole body was under this morbid influence, she was placed in a standing position, with her limbs in various attitudes, which would with difficulty have been assumed, even for a moment, by a person in health, and which certainly could not voluntarily have been supported for so long a time as she remained fixed without the slightest motion. When the muscles were no longer under the morbific influence, or supplied by this irregular distribution of the nervous power, the parts which had been previously extended, or placed in any particular position, suddenly dropped upon the bed in a state of relaxation. From a standing or sitting position, she fell like a person struck down with great violence, and then for some time remained motionless as before. It would be uninteresting to dwell minutely upon the various attacks she had of a similar nature. She continued in this state for about a fortnight, with occasional intervals, during which her mental faculties appeared perfect, but were with difficulty brought into action.

For two or three days afterwards, her malady assumed the form of common chorea. A complete alteration of symptoms then occurred. She was free from convulsive action, and almost completely regained her mental activity. She now complained of great pain in her breasts, which were obviously enlarged, and their whole surface was remarkably hard and tense. The axillary glands were also slightly enlarged. Some warm fomentations were applied, and the breasts in the course of the next day assumed their natural appearance.

During the whole progress of the case, every means were adopted to regulate the state of the bowels. This was always accomplished with difficulty, but it may be observed that, at the time of her becoming cataleptic, she had daily evacuations, of a natural appearance.

She was afterwards removed to St. George's Hospital, and during her short stay there I understand the same curious alternation of symptoms occurred, together with a return of the cataleptic state. She is now in the country, in a very debilitated state of health, and still suffers from violent attacks of hysteria. Two issues have been inserted in her neck and arm, without any alleviation of her symptoms.

The anomalous and inexplicable+ symptoms which frequently occur in hysterical females are too well known to require any

+ not at all — the cause & the remedy are plain enough particular comment; and I am aware that the experienced physician would not participate in the alarm which is always excited by that death-like appearance I have here described, and which so frequently succeeds to violent paroxysms of hysteria. It is now nearly eighteen years ago since I first saw a patient in this apparently hopeless state, and I confess, without hesitation, that I imagined, and declared to the friends, that death would speedily ensue. In many cases which I have subsequently seen, I have known young men compromise their reputation, as I had done, by giving a decidedly unfavourable prognosis, when a more experienced practitioner has declared, without hesitation, that the life of the patient was not in danger. We should not then forget the observation of HOFFMAN upon the subject of hysteria: "Ut valdé terribilis hic videtur morbus, in se tamen non adeò periculosus est." The caution I wish to convey upon this subject can only be necessary for those who are upon the threshold of their professional avocations, and it is to such only that I presume to direct my observations.

Hysteria, of course, like every other disease, may be complicated with some other affection of a more dangerous tendency, and our prognosis must then be modified accordingly. Neither must it be forgotten that confirmed epilepsy, mania, and other very serious consequences, sometimes result from repeated attacks of simple hysteria. But I have neither seen nor heard of any cases of women having died in that state of lethargy and total insensibility which so frequently occurs after violent hysterical paroxysms.

During the progress of hysterical complaints, symptoms of local inflammation not unfrequently occur, which are also likely to deceive the inexperienced practitioner. I believe, however, with Dr. MARSHALL HALL, that it very rarely happens that "attacks of hysteria concur with the active inflammation of a vital organ," although nothing is more common in practice than to bleed largely in such cases, from an apprehension of organic disease. It will be found, however, that the acute local pain, of which hysterical women very frequently complain, will speedily subside in most cases without the use of the lancet, or at least by a very moderate abstraction of blood. The pain may be severe for a few hours, but it is rarely fixed. The hysterical patient often complains of intense pain of the head, of the chest, and of the side, in rapid succession. There is scarcely an organ of the body which does not occasionally suffer in the course of this ever-varying disease, but still the symptoms are very different from those of pure local inflammation, although they are frequently supposed to indicate its existence, and to lead to a mode of treatment which is at once unnecessary and prejudicial to the patient. Upon this subject I beg particularly to refer to the ninth chapter of Dr. Hall's recent publication.* I have seen many cases which convince me of the accuracy and judgment of his remarks. In hysterical women there is also very frequently a tenderness of the whole surface of the body, which confirms the suspicion of local inflammation. When there is this state of morbid sensibility to the slightest pressure, the patient will equally complain whether it is applied to the part which has been previously the seat of pain, or to any other part of the body. Several instances of this sort have occurred to me in the course of the last few years.

It is necessary, then, that we should not hastily infer the existence of idiopathic inflammatory disease from the occurrence of the wandering pains of various parts, to which hysterical women are subject. These complaints may, perhaps, be more correctly attributed to an irregular distribution of the nervous influence, than to any inflammatory affection. A constipated state of the bowels is much more common in females than in men. In many instances I have known women, apparently in good health, who had seldom more than one evacuation every third day. To this irregular condition of the digestive organs but little attention is paid, and there can be no doubt that it frequently lays the foundation for hysteria and other nervous affections.

---

### RETENTION OF URINE.

* Dr. Marshall Hall on some Diseases of Females: 1827.

